# Present to Prof. Davis

**Published:** 1853-10

**Authors:** 


An Elegant Present Worthily Bestowed.—The class of Medical Students attending the last session of Rush Medical College, have presented Professor Davis a very valuable present, as an evidence of their appreciation of his ability and faithfulness as a teacher; consisting of a *Microscope,* supplied with three achromatic object glasses of the best quality, one of which affords a magnifying power equal to 1,000 diameters. On the front of the case in which the tube slides is neatly engraved the following:

Presented
to
N. S. DAVIS, M. D.,
Prof. of Pathology and Practice
of
Medicine in
Rush Medical College,
By the Class of
1852–3.

The class have displayed both good taste and liberality in selecting so appropriate and valuable a gift. Professor Davis ranks high in his profession, while his course as a private citizen has attracted around him a large number of personal friends.

---

Cards.—The attention of our medical brethren is called to the cards of D. Kolbe and Keumerele, manufactures of surgical instrstruments and other appliances. Mr. Kolbe is an artist of superior qualifiactions in his department, having been taught in Paris, France, to form and fashion the most delicate instrument. Keumerle has on hand and is prepared to manufacture to order, every instrument in the form of pump or syringe, and all instruments required to be turned from ivory or wood. The card of Hartman and Saxe speaks only of mathematical instruments, but the superiority of his optical and other instruments required by the profession, deserves special attention and notice. Specimens of the superior workmanship of all these gentlemen can be seen by calling upon the Dean of the Faculty, he having supplied himself liberally from each.

---

Dr. Marshall Hall, who was lately in Boston, intends to proceed to New Orleans, for the purpose of witnessing the surprising experiments of Drs. Cartwright and Dowler, on alligators. Dr. H. is much interested in the physiological researches with reference to the nervous system, lately instituted in connection with these startling experiments.

